# Sweet’s syndrome associated with FLT3 inhibition

**DOI:** 10.1016/j.jdcr.2024.09.013

**Published:** 2024-09-30

**Authors:** Riyad N.H. Seervai, Elena Paz Munoz, S. Caleb Freeman, Ronan I. Swords, Kevin P. White, Jesse J. Keller

**Affiliations:** a Department of Dermatology, Oregon Health & Science University, Portland, Oregon; b Division of Hematology/Oncology, Department of Medicine, Knight Cancer Institute, Oregon Health & Science University, Portland, Oregon

**Keywords:** cutaneous toxicity, FLT3 kinase inhibitor, gilteritinib, neutrophilic dermatosis, oncodermatology, quizartinib, Sweet’s syndrome

## Introduction

Internal tandem duplication mutations in the fms-like tyrosine kinase 3 (*FLT3*) gene have been identified in ∼30% of relapsed/refractory acute myeloid leukemia (R/R AML) patients and have been associated with more aggressive disease and worse survival.^1^ The FLT3 inhibitor gilteritinib has been shown to prolong survival and promote remission in these patients.^2^ Cutaneous toxicities associated with gilteritinib include a nonspecific “rash” in up to 30% of patients^3^ and rare cases of neutrophilic dermatoses. Here, we report a case of Sweet’s syndrome (SS) associated with gilteritinib that improved with cessation and reoccurred with rechallenge of this and quizartinib, providing a clearer link between this neutrophilic dermatosis and FLT3 inhibition. SS might be a toxicity associated with a class effect and could limit the use of FLT3 inhibitors in some patients with R/R AML.

## Case presentation

**Fig 1:**
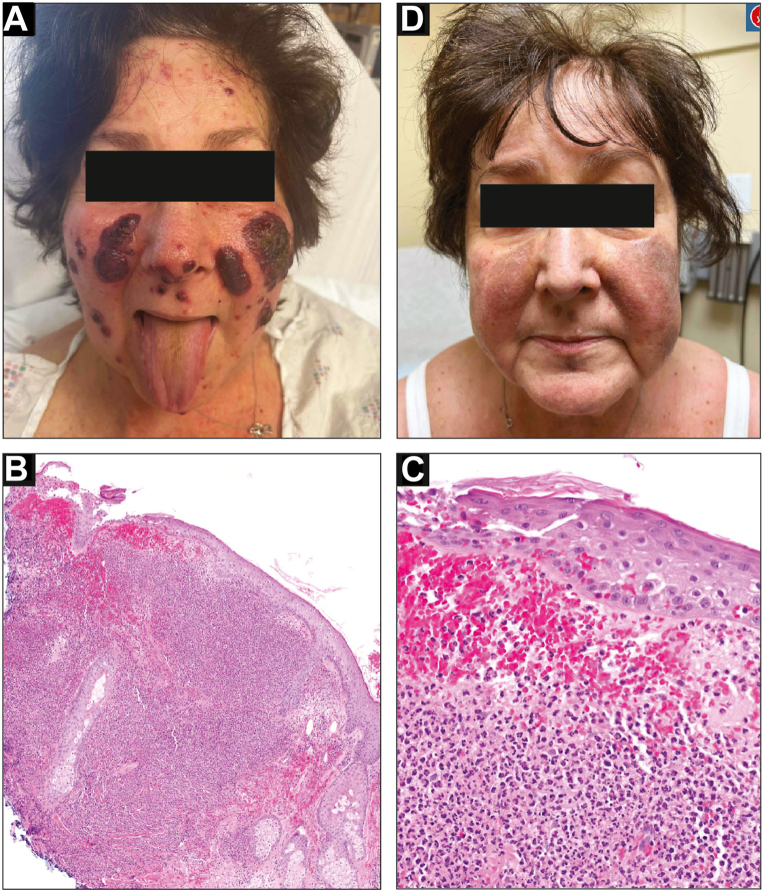
Initial presentation of Sweet's syndrome with gilteritinib. **A,** Well-demarcated, edematous, violaceous plaques on nose and cheeks with yellow-green crust and pustulation. **B** and **C,** Histopathology showing epidermal erosion, erythrocyte extravasation, and dense nodular neutrophilic infiltrate. Hematoxylin and eosin, original magnification ×4 (**B**) and ×20 (**C**). **D,** Resolution of plaques with prednisone treatment at 4-week follow-up.

The patient is a 73-year-old woman with a history of R/R AML with multiple mutations including in *FLT3*. She failed traditional chemotherapy and was initiated on combination therapy with the lysine-specific demethylase-1 inhibitor iadademstat and gilteritinib as part of the FRIDA trial (NCT05546580). Twenty-five days after initiation, she presented with fever and a new rash. Laboratory workup was notable for normal white blood cell count and negative infectious workup. Exam showed multiple coalescing, sharply-demarcated, edematous, and violaceous papules and plaques on the nose and cheeks, some with yellow-green crust and pustulation (Fig 1, *A*). There was no oral or ocular involvement. Biopsy showed epidermal erosion with a dense nodular neutrophilic infiltrate (Fig 1, *B* and *C*). Periodic acid-Schiff, Fite, and Gram stains did not reveal microorganisms, and her bacterial/fungal blood cultures and viral workup were negative. Collectively, this prompted a diagnosis of SS. She was started on prednisone at 50 mg (0.75 mg/kg) daily with a plan for taper, with rapid resolution of her symptoms (Fig 1, *D*).

**Fig 2:**
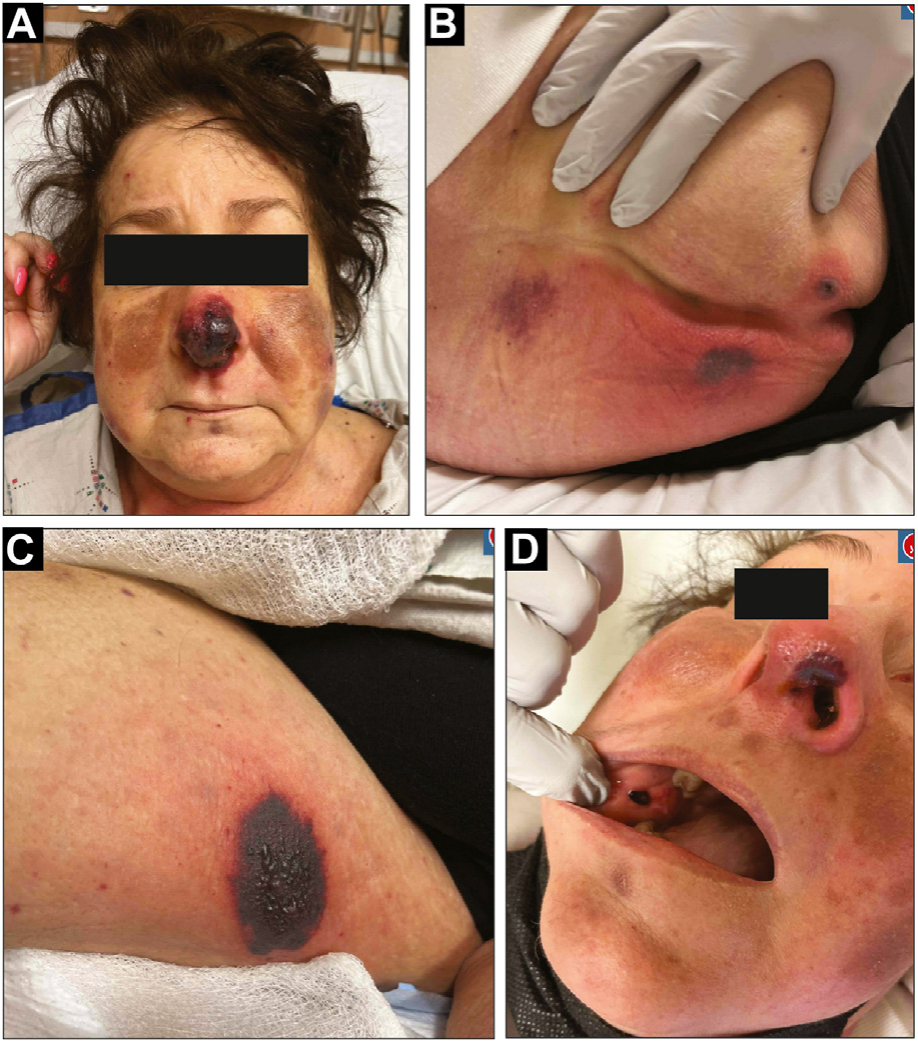
Reoccurrence of Sweet's Syndrome with gilteritinib rechallenge. Well-demarcated, violaceous plaques on the nose (**A**), buttocks (**B**), and right inner thigh (**C**). **D,***Dark red* to *purple* thin plaque on the right buccal mucosa.

A decision was made to rechallenge with gilteritinib at a reduced dose (80 mg from 120 mg) given her rapid response to the prednisone. However, 8 days later, she presented with fever, chills, and recurrent lesions on the nose, buttocks, and right inner thigh (Fig 2, *A*-*C*). She also reported oral involvement, with exam showing a dark red to purple thin plaque on the buccal mucosa (Fig 2, *D*). Labs were significant for neutropenia, hypokalemia, and hyperbilirubinemia. The lesions were consistent with SS reoccurrence. Bone marrow biopsy at this time showed improvement in her AML, with neutrophil recovery and <5% blasts but with persistent *FLT3* mutation. Her prednisone was increased to 50 mg daily, her symptoms rapidly resolved, and gilteritinib was permanently discontinued.

**Fig 3:**
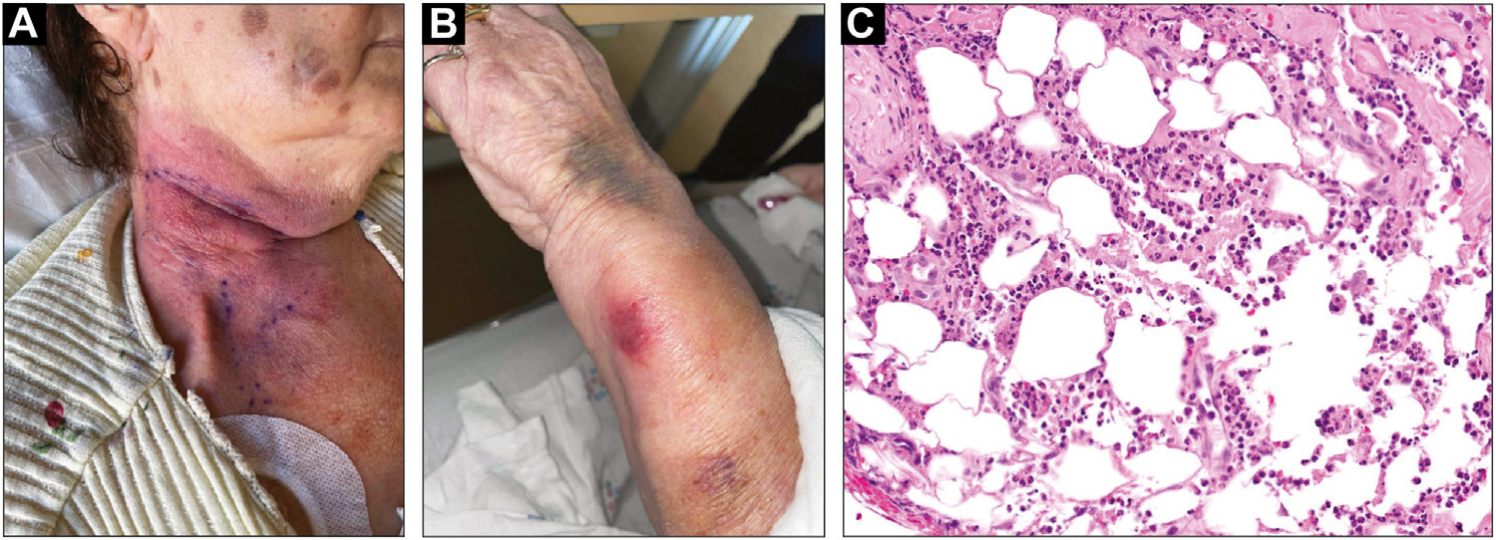
Reoccurrence of Sweet's syndrome with quizartinib challenge. **A,** Well-demarcated, indurated *pink* plaque on the right neck. **B,** Ill-defined, edematous, round *red* plaque on the left wrist. **C,** Histopathology showing diffuse suppurative dermatitis and neutrophilic panniculitis. Hematoxylin and eosin, original magnification ×20.

The patient presented back to the hospital following a fall with new visual symptoms. Lumbar puncture showed central nervous system involvement with significantly elevated atypical white blood cells in the cerebrospinal fluid. A decision was made to trial her on the alternative FLT3 inhibitor quizartinib. Three weeks later, she presented with a tender indurated pink plaque on the right neck and an ill-defined round red edematous plaque on the left wrist that did not respond to broad-spectrum antibiotics (Fig 3, *A* and *B*). Biopsy of the neck lesion showed a diffuse neutrophilic and lymphocytic infiltrate extending to areas of fat necrosis (Fig 3, *C*). Infectious workup (special stains, blood/fungal cultures, viral swabs) was negative. Dermatology recommended prednisone given high suspicion for recurrent SS, and her symptoms again resolved. She underwent rapid deterioration; the quizartinib was discontinued due to difficulty swallowing, and the patient ultimately died from AML progression.

## Discussion

Our patient’s SS posed a diagnostic and therapeutic conundrum for her treatment team. SS is classified as idiopathic (classical), drug-induced, or malignancy-associated. AML is the most common malignancy associated with SS,^4^ and an SS-like neutrophilic infiltrate has been reported as the initial presentation of AML.^5^ Granulocyte colony-stimulating factor treatments such as filgrastim are the most common drugs associated with a drug-induced SS in these patients.^4^ There are no reports of iadademstat as a cause of SS.^6^ However, gilteritinib and other FLT3 inhibitors have been implicated in several reports of SS and other neutrophilic dermatoses.^7^^,^^8^

Induction of terminal myeloid differentiation in arrested bone marrow precursors with FLT3 inhibition has been implicated in the pathogenesis of FLT3 inhibitor-associated SS and neutrophilic dermatoses. Fathi and colleagues^8^ described a “dermatologic differentiation syndrome” in which quizartinib-induced neutrophilic dermatosis samples contained neutrophils that originated from *FLT3*-mutant precursor myeloblasts. Similar differentiation syndromes with SS have been described during treatment with the isocitrate dehydrogenase-1 inhibitor ivosidenib for R/R AML and all-trans retinoic acid for acute promyelocytic leukemia.

We used the Naranjo adverse drug reaction (ADR) probability scale^9^ to estimate the likelihood the patient’s SS was related to FLT3 inhibitor therapy (Table I). At the time of decision to rechallenge with gilteritinib at a lower dose, her initial reaction was thought to be a “possible” ADR based on temporality and improvement with interruption. She fulfilled all 5 Walker and Cohen criteria for drug-induced SS,^10^ and reoccurrence of her symptoms with rechallenge suggested a “definite” ADR leading to permanent discontinuation of the gilteritinib. When she later presented with central nervous system involvement, she was trialed on the alternative FLT3 inhibitor quizartinib and her final dermatologic symptoms again favored SS as a “possible” ADR. Although we strongly favor FLT3 inhibition as a cause of her SS, her underlying AML casts doubt on this as the sole contributor. While bone marrow biopsy showed improvement in her AML at the time of rechallenge with gilteritinib, her subsequent central nervous system symptoms and death indicate an overall progression of the disease. We cannot exclude the possibility her symptoms represented malignancy-associated SS that improved with prednisone and flared when this was tapered, and that FLT3 inhibition had a secondary role.

**Table I:** Naranjo adverse drug reaction probability scores for patients’ episodes of Sweet’s syndrome, calculated at the time of decision to discontinue vs rechallenge

	Initial presentation with gilteritinib	Reoccurrence with gilteritinib rechallenge	Reoccurence with quizartinib trial
Previous conclusive reports about reaction	Yes (+1)	Yes (+1)	Yes (+1)
Adverse event appeared after drug was given	Yes (+2)	Yes (+2)	Yes (+2)
Reaction improved when drug was discontinued	Yes (+1)	Yes (+1)	Not known (0)
Reaction appeared when drug was readministered	Not known (0)	Yes (+2)	Not known (0)
Alternative causes that could have caused the reaction	Yes (−1)	No (+2)	Yes (−1)
Reaction reappeared when placebo given	Not known (0)	Not known (0)	Not known (0)
Drug detected in any body fluid in toxic concentrations	Not known (0)	Not known (0)	Not known (0)
Reaction more severe with increased dose, less severe with decreased dose	No (0)	No (0)	Not known (0)
Similar reaction to same or similar drugs in any previous exposure	No (0)	Yes (+1)	Yes (+1)
Adverse event confirmed by any objective evidence	Yes (+1)	Yes (+1)	Yes (+1)
Score	4 (possible)	10 (definite)	4 (possible)

Total scores range from −4 to +13. Doubtful reaction (−4 to 0) is likely related to other factors. Possible reaction (1 to 4) follows a reasonable temporal sequence, possibly follows a recognized pattern, and could be explained by other factors. Probable reaction (5-8) follows a reasonable temporal sequence, follows a recognized pattern, is confirmed by improvement on withdrawal of the drug, and is not reasonably explained by other factors. Definite reaction (9+) follows a reasonable temporal sequence, follows a recognized pattern, and is confirmed by improvement on withdrawal and reappearance on reexposure.

In summary, our case highlights the complexity of considering FLT3 inhibitors as a cause of drug-induced SS in the setting of AML or other potential confounders. In many cases, AML progression is the primary driver of the neutrophilic dermatosis. For some patients, SS might represent a class effect that limits use of other FLT3 inhibitors for their AML. Therapy is generally interrupted regardless of whether a better alternative cause is feasible or not, and rechallenge can potentially elucidate the etiology of malignancy-associated versus drug-induced SS. All these factors can independently and synergistically contribute to the underlying pathophysiology of SS, and the uncertainty in pinpointing a specific cause reflects the real-world challenges of supportive oncodermatology. A multidisciplinary approach can help alleviate some of this uncertainty, and additional work is needed on how to best serve these patients at these inflection points during their anticancer therapy.

## Conflicts of interest

None disclosed.
